# Integrin α2β1 plays an important role in the interaction between human articular cartilage-derived chondrocytes and atelocollagen gel

**DOI:** 10.1038/s41598-021-81378-2

**Published:** 2021-01-19

**Authors:** Takashi Kanamoto, Minami Hikida, Seira Sato, Shohei Oyama, Yoshihito Tachi, Sanae Kuroda, Takeo Mazuka, Kosuke Ebina, Tsuyoshi Nakai, Ken Nakata

**Affiliations:** 1 Department of Medicine for Sports and Performing Arts, Osaka University Graduate School of Medicine, 2-2 Yamadaoka, Suita, Osaka 565-0871 Japan; 2 Department of Musculoskeletal Regenerative Medicine, Osaka University Graduate School of Medicine, 2-2 Yamadaoka, Suita, Osaka 565-0871 Japan; 3 Olympus-RMS CORP, 3-1-7 Myojin-cho, Hachioji, Tokyo 1920046 Japan; 4 Department of Orthopedic Surgery, Itami City Hospital, 1-100 Koyaike, Itami, Hyogo 664-8540 Japan; 5 Department of Orthopedic Surgery, Hannan Chuo Hospital, 3-3-28 Minami-shinmachi, Matsubara, Osaka 580-0023 Japan

**Keywords:** Cartilage, Osteoarthritis, Biomaterials

## Abstract

Although atelocollagen gel is used as a scaffold for culturing human articular cartilage-derived chondrocytes, little is known about cell–gel interactions. In this study, we investigated the mechanism via which atelocollagen gel affects human articular cartilage-derived chondrocytes. Two types of three-dimensional cultures of human articular cartilage-derived chondrocytes (i.e., with and without atelocollagen gel) were compared. While the amount of atelocollagen gel in culture gradually decreased with time, it promoted the expression of matrix metalloproteinases (MMPs) during the early stages of culture. Genome-wide differential gene expression analysis revealed that cell membrane- and extracellular matrix-related genes were highly ranked among up- and down-regulated groups in cells cultured in the presence of atelocollagen gel. Among the integrin family of genes, the expression of integrin subunit alpha 2 and integrin subunit alpha 10 was significantly increased in the presence of atelocollagen gel. Blocking α2β1 integrin with the specific inhibitor BTT 3033 had a significant effect on cell proliferation, MMP expression, and cell shape, as well as on the response to mechanical stimulation. Taken together, our findings indicate that the α2β1 integrin pathway plays an important role in the interaction of atelocollagen gel with human articular cartilage-derived chondrocytes and may be a potential therapeutic target for articular cartilage disorders.

## Introduction

Knee articular cartilage disorder can result from sports injury, for example, and osteoarthritis (OA) represents an important issue in society and medicine^1, 2^. Osteoarthritis is the most common joint disorder, affecting more than 10% of people over 60 years of age worldwide, and the resultant socioeconomic impact due to the aging population is increasing. Articular cartilage consists of a limited number of chondrocytes and an abundant extracellular matrix (ECM), which contains high levels of aggregating proteoglycan aggrecan and type 2 collagen containing heterotypic collagen fibrils^3^. Thus, understanding the interactions between cells and the ECM, as well as their behaviors and characteristics, is important for developing more effective treatments for a damaged or degenerated articular cartilage^4^. You cannot alter accepted Supplementary Information files except for critical changes to scientific content. If you do resupply any files, please also provide a brief (but complete) list of changes. If these are not considered scientific changes, any altered Supplementary files will not be used, only the originally accepted version will be published.We have checked AQ1 and agreed.

The use of autologous chondrocyte implantation (ACI) in humans was first reported in 1987 and is now an established treatment for articular cartilage disorders of the knee. ACI involves culturing cartilage-producing cells from the knee articular cartilage and implanting them into the chondral defect^5, 6^. The original ACI technique involved the injection of a suspension of cultured chondrocytes into a debrided chondral defect under a periosteal cap, and this procedure has evolved over time^5, 7^. Second-generation ACI used a collagen cap instead of a periosteal cap. In third-generation ACI, chondrocytes are loaded, embedded, or seeded onto a collagen membrane. Although these technical developments helped to establish ACI as a cell therapy, key issues, including a limited source of cells and chondrocyte de-differentiation, remain unresolved^8, 9^. Several new procedures have been devised to address these issues, and their therapeutic effects are currently under investigation^10, 11^.

Collagen, the most abundant protein in the human body, plays an important role in the formation of tissues and organs and is involved in various cellular functions^12^. Specific cell receptors and protein domains play dominant roles not only in cell connection and migration, but also in the regulation and stimulation of cell differentiation and specific protein expression at the gene level. Given its highly desirable characteristics, such as biodegradability and safety, the use of collagen in the field of tissue engineering has been steadily increasing^13^. Among collagen-based biomaterials, atelocollagen is produced by removing a telopeptide that retains the antigenicity from type 1 collagen. Atelocollagen has been used clinically as a scaffold/cell carrier for regenerative medicine in many fields, for example, in the treatment of articular cartilage disorders^11, 14^. A prospective multicenter clinical trial of atelocollagen-associated chondrocyte implantation has showed good clinical outcomes. The authors concluded that transplanting chondrocytes in a newly formed matrix of atelocollagen gel can restore the articular cartilage of the knee^15^.

Three-dimensional (3D) culture of human articular cartilage-derived chondrocytes using collagen gels, such as atelocollagen gel, has been studied for a while^16, 17^. Numerous studies have addressed the effects of collagen gel on cell proliferation, cell differentiation, and substrate production, and its usefulness as a scaffold is widely recognized. Microenvironments are important for various biological functions, with recent reports demonstrating the importance of cell–ECM interactions in stem cell fate determination and chondrocyte phenotype^18, 19^. However, limited information is available regarding the interactions between atelocollagen gel and human articular cartilage-derived chondrocytes, and the mechanism via which atelocollagen gel affects these cells. Thus, further elucidation of the impact of these interactions may have therapeutic implications. To this end, the present study aimed to examine the interactions between human articular cartilage-derived chondrocytes and atelocollagen gel, and their impact on cell proliferation, gene expression, and on the response to mechanical stimulation in 3D cultures with and without atelocollagen.

## Results

### Collagen gel affects the metabolic activity, but not the proliferation, of human articular cartilage-derived chondrocytes in 3D culture, as well as the expression of cell cycle-related factors and chondrogenic differentiation markers

**Figure 1:**
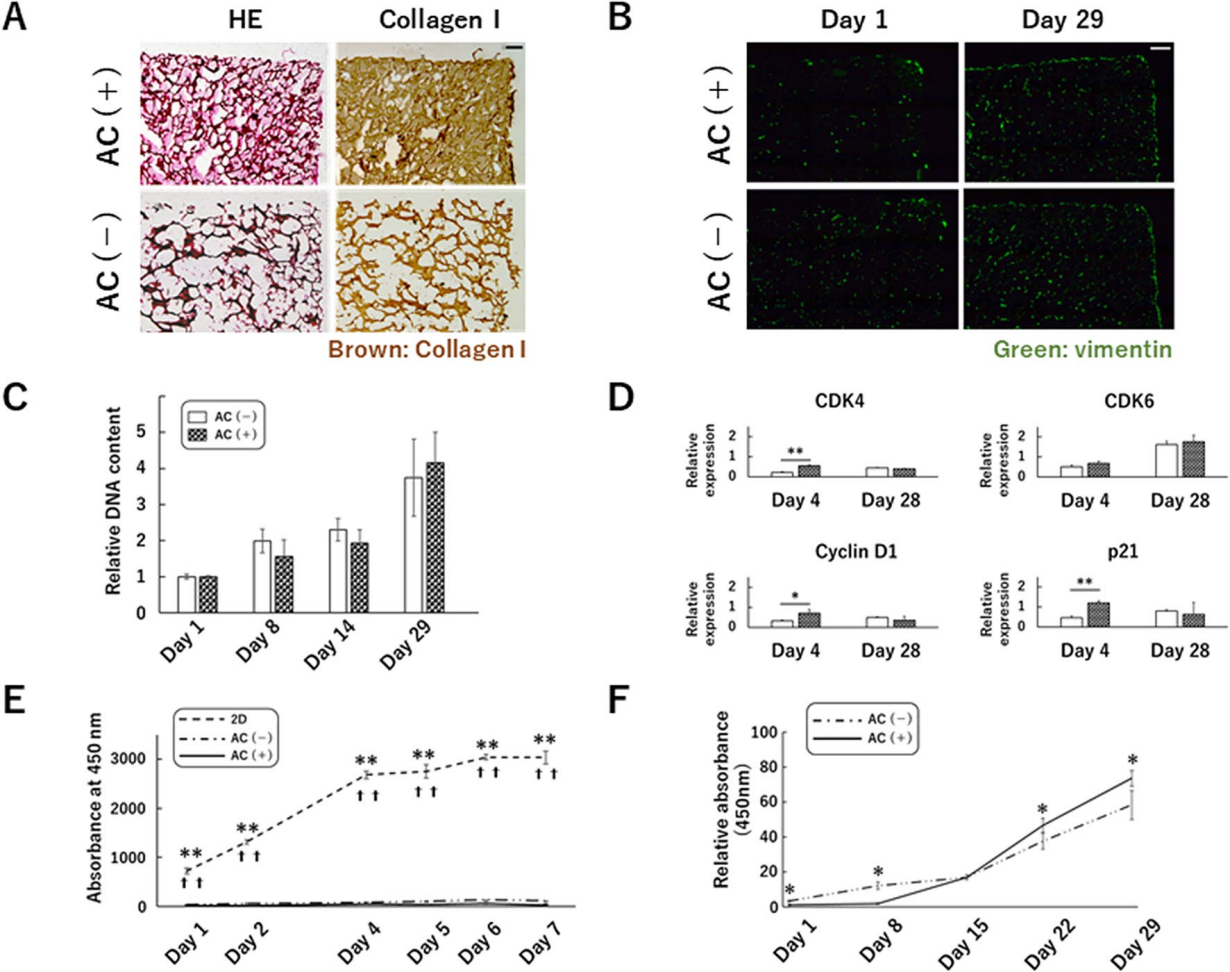
Effects of atelocollagen gel on human articular cartilage-derived chondrocytes in three-dimensional culture. (**A**) Semi-serial sections were stained with hematoxylin and eosin and immunostained with anti-type I collagen antibody. Atelocollagen gel (brown signal) is observed in the pores of the entire sponge (upper right panel). Scale bar: 200 μm. (**B**) Sections immunostained with anti-vimentin antibody. Green: Vimentin. Scale bar: 200 μm. (**C**) Changes in the amount of cellular DNA in the sponge over time. Total DNA content is measured on day1, 8, 14, and 29. Data are normalized to the values at day1. (**D**) Comparison of the expression of cell cycle-related factors. Normalized expression relative to 2D culture cells at approximately 50–60% confluency (semi-confluent monolayers) is shown. (**E**,**F**) The metabolic activity after seeding 5000 cells / well or sponge was evaluated over time by absolute absorbance using a Cell Counting Kit-8. (**E**) Comparison between 2D culture, AC(−), and AC(+) groups for 7 days. ** and ✝✝denote statistically significant differences between 2D culture and AC(−) group or between 2D culture and AC(+) group (*p* < 0.01), respectively. (**F**) Comparison between the AC(−) and AC(+) groups for 29 days. The relative absorbance is shown with the value in AC(+) on day 1 as 1. (**C**,**D**,**E**,**F**) A representation of three independent experiments, each with three subjects, is shown. *p < 0.05, **p < 0.01.

We first examined the distribution of atelocollagen gel in atelocollagen sponge immediately after cell seeding by staining with anti-collagen type I antibody. The continuous pores of the sponge were filled with gel in the group using atelocollagen gel [AC(+) group; Fig. 1A]. Following cell seeding, 3D culture was continued in growth medium and cells were found throughout the sponge on day 29 of culture in both the AC(+) group and the group that did not use atelocollagen gel [AC(−) group] (Fig. 1B). DNA content increased during the first 4 weeks of culture, and no significant differences were observed with or without atelocollagen gel (Fig. 1C). Moreover, the expression of cell cycle-related factors (CDK4*,* Cyclin D1, p21) was higher in the AC(+) group than in the AC(−) group on day 4 of culture (Fig. 1D). Comparing 2D and 3D cultures of cell proliferation, the cell metabolic activity in the 2D culture was significantly higher during the week after cell seeding (Fig. 1E). Metabolic activity was significantly lower in the AC(+) group than in the AC(−) group in the early stages of culture, but significantly higher on days 22 and 29 of culture (Fig. 1F).

### Expression of multiple MMPs increases at an early stage of culture, but gradually decreases with time, in the presence of atelocollagen gel

**Figure 2:**
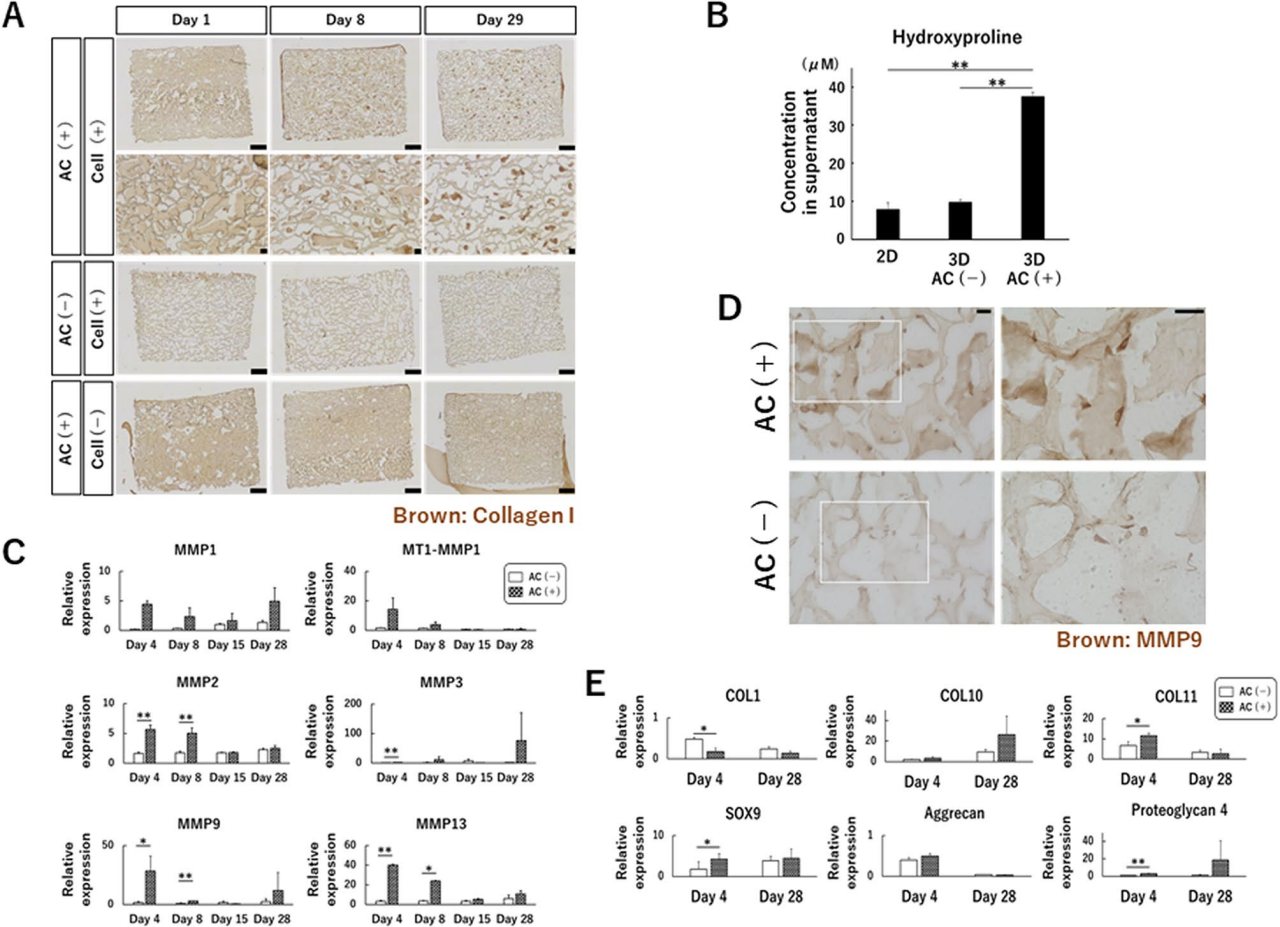
Atelocollagen gel impacts the expression of MMP family members. (**A**) Sections prepared from three different samples on three time points. AC(+), AC(−), and atelocollagen sponge without cells were immunostained with anti-type I collagen antibody. Scale bar: 200 μm. (**B**) The concentration of hydroxyproline in the supernatant was determined on day 3 of culture, as described in the Materials and Methods. (**C**) RT-qPCR analysis of MMPs. Normalized expression relative to 2D culture cells is shown. (**D**) Representative images of immunostaining using anti-MMP9 antibody in AC(−) and AC(+) samples. Right panels are magnifications of the square regions in the left panels. (**E**) Comparison of the expression of factors related to chondrocyte differentiation. (**C**,**E**) A representation of three independent experiments, each with three subjects and normalized expression relative to 2D culture cells at approximately 50–60% confluency (semi-confluent monolayers) is shown. *p < 0.05, **p < 0.01.

Atelocollagen is biodegradable. When the distribution of the gel in 3D culture was examined over time, the area occupied by atelocollagen gel gradually decreased with time (Fig. 2A). When cells were cultured with atelocollagen gel, the brown signal (corresponding to atelocollagen gel) decreased as the duration of culture increased. On day 29 of culture, dark brown spots were observed, which indicated the contraction and degradation of the gel. No change was observed in the absence of cells. When hydroxyproline in the culture supernatant was quantified for the purpose of examining collagen degradation, a significantly larger amount of product was detected in the AC(+) group compared to the 2D culture and AC(−) groups (Fig. 2B). Next, we examined the expression of several matrix metalloproteinase (MMP) family members, which are candidate collagen-degrading enzymes. The expression of MMP2, MMP3, MMP9, and MMP13 was significantly higher in the AC(+) group than in the AC(−) group on day 4 of culture, but gradually decreased with culture time (Fig. 2C). Immunostaining of the samples on day 4 of culture showed a clear expression of MMP9 in the AC(+) group, while it showed an unclear expression in the AC (−) group (Fig. 2D). No other MMPs (MMP1, MMP2, and MMP13) showed a clear expression in the AC(+) and AC(−) groups (Fig. S1). The expression of chondrogenic differentiation markers (*collagen 1*, *collagen 11*, *sox9*, and *proteoglycan 4*) also differed between the AC(+) and AC(−) groups on day 4, but did not significantly differ on day 29 (Fig. 2E). The expression of chondrocyte hypertrophic markers and hypoxia-inducible factors also showed a significant difference between the AC(+) and AC(−) groups on day 4 (Fig. S2).

### Atelocollagen gel regulates the expression of cell membrane- and extracellular matrix-related genes and promotes the expression of* ITGA2* and *ITGA10* in 3D cultures

**Figure 3:**
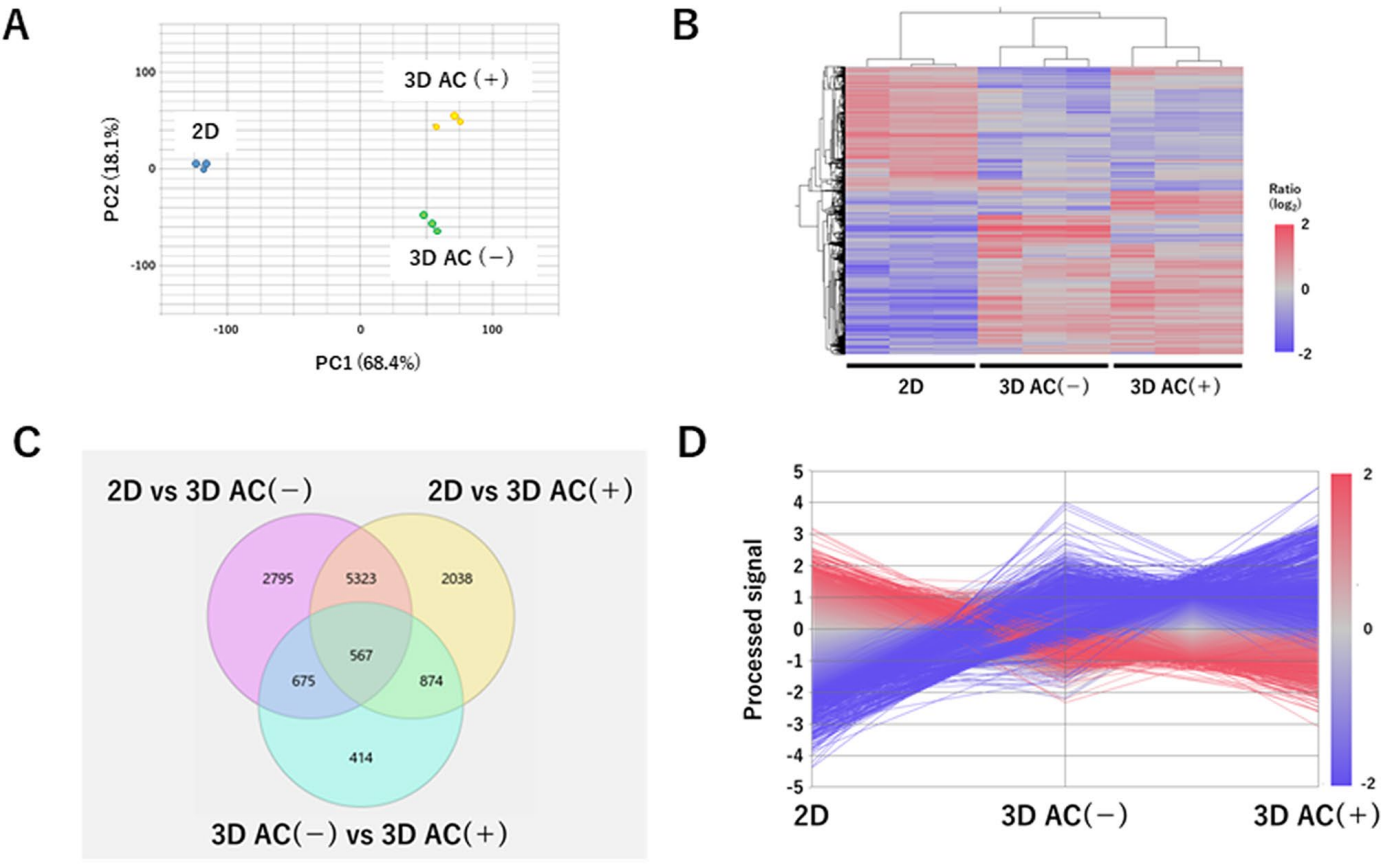
Gene expression in human articular cartilage-derived chondrocytes using a culture method. (**A**) Principal component analysis (PCA) of regularized-logarithm transformed read counts, in which three culture method groups were separated along the axis of major variation (PC1, 68% of variance). 2D: 2-dimensional culture, 3D: 3-dimensional culture. (**B**) Hierarchical clustering of 16,586 genes whose transcript levels changed by more than twofold or less than ½-fold, depending on the culture method. (**C**) Overlap of differentially expressed genes (DEGs) in the three comparisons: 2D culture versus 3D culture without atelocollagen gel, 2D culture versus 3D culture with atelocollagen gel, and 3D culture without atelocollagen gel versus 3D culture with atelocollagen gel. (**D**) Line graph representation of the transcript levels of genes shown in (**B**). The mapped data were analyzed using the Subio platform version 1.24 (Subio, Inc., Kagoshima, Japan, http://www.subio.jp).

**Figure 4:**
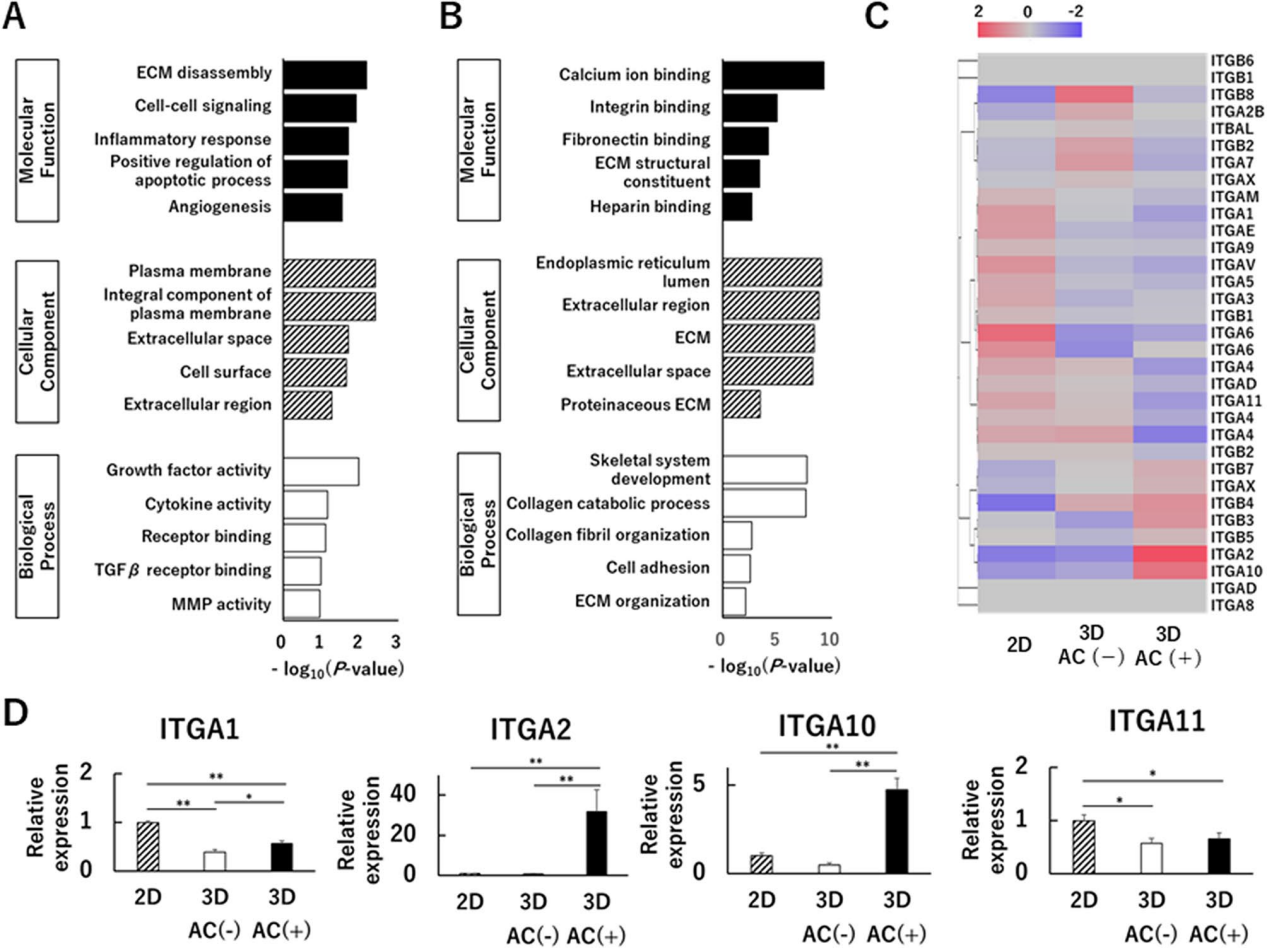
Atelocollagen gel significantly affects the expression of cell membrane- and extracellular matrix-related genes. (**A**,**B**) Gene ontology (GO) enrichment analysis comparing highly ranked (|log 2 FC|> 2) differentially expressed genes (DEGs) in 3D culture with atelocollagen gel and 3D culture without atelocollagen gel. (**A**) Enriched GO terms for the 1,863 genes upregulated in 3D culture with atelocollagen gel. (**B**) Enriched GO terms for the 2,056 genes down-regulated in 3D culture with atelocollagen gel. BP, biological process; CC, cellular component; MF, molecular function. (**C**) Heat map (logarithmic expression) of integrin family member genes presented in a mean enrichment plot across three culture groups. The mapped data were analyzed using the Subio platform version 1.24 (Subio, Inc., Kagoshima, Japan, http://www.subio.jp). (**D**) Comparison of expression of collagen-binding integrins. A representation of three independent experiments, each with three subjects and normalized expression relative to 2D culture cells at approximately 50–60% confluency (semi-confluent monolayers) is shown. *p < 0.05, **p < 0.01.

Genome-wide differential gene expression analysis was performed to examine the effects of atelocollagen on cultured cells. Biological replicates of the same culture conditions were clustered together using principal component analysis and cluster analysis (Fig. 3A,B). There were substantial differences in the gene expression patterns between 2 and 3D cultures. In addition, the presence of atelocollagen gel significantly affected gene expression in 3D cultures (Fig. 3C,D). In the enrichment analysis using the highest rank (|log2 FC|> 2) of differentially expressed genes (DEGs) comparing 3D cultures of the AC(+) and AC(−) groups, extracellular matrix-related factors were categorized as the highest in each category (BP for Biological Process, MF for Molecular Function, and CC for Cellular Component) of the up-/down-regulated groups (Fig. 4A,B). Among the molecules that bind to collagen as part of the extracellular matrix, we focused on the integrin family. Several genes, including integrin subunit alpha 2 (*ITGA2*) and integrin subunit alpha 10 (*ITGA10*), were found to be highly expressed in the AC(+) group and to have a low expression in the AC(−) group (Fig. 4C). The measurement of the expression of four genes (*ITGA1*, *ITGA2*, *ITGA10*, and *ITGA11*) encoding collagen-binding integrins using qPCR revealed that the expression of *ITGA2* and *ITGA10* was higher in the AC(+) group than in the AC(−) group and 2D cultures (Fig. 4D).

### BTT 3033, an α2β1 integrin-specific inhibitor, affects proliferation, MMP expression, and cell shape in the AC(+) group

**Figure 5:**
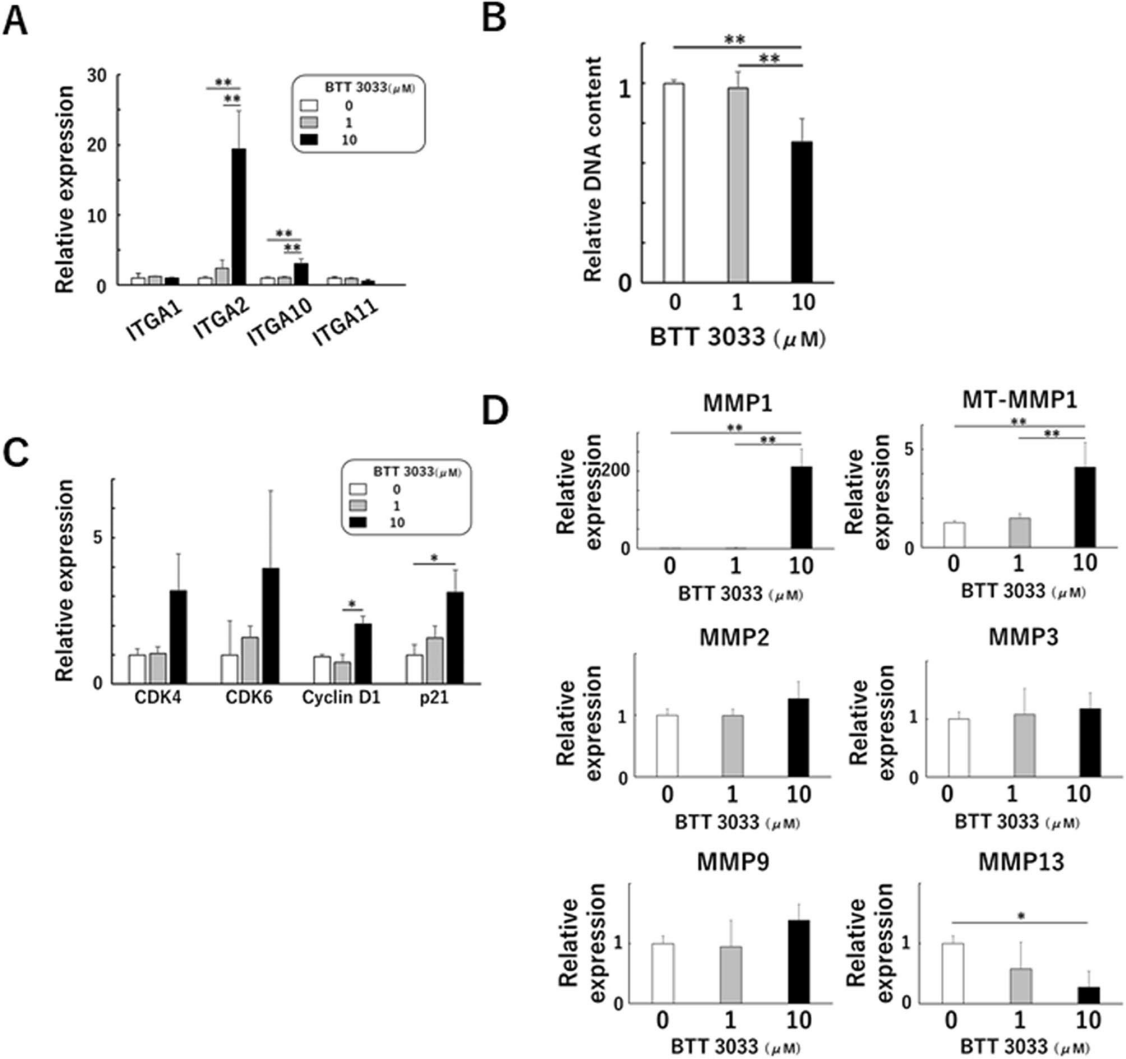
BTT 3033, a selective integrin inhibitor alters cell proliferation and MMP expression in AC(+) group. Expression of collagen-binding integrins (**A**), cell cycle-related factors (**C**), and MMP family (**D**) in the presence of BTT 3033, a selective inhibitor of α2β1 integrin, in cells cultured with atelocollagen gel. Normalized expression relative to cells cultured in the absence of BTT 3033 is shown (**A**,**C**,**D**). (**B**) DNA content relative to cells cultured in the absence of BTT 3033 is shown. A representation of three independent experiments, each with three subjects is shown. *p < 0.05, **p < 0.01.

**Figure 6:**
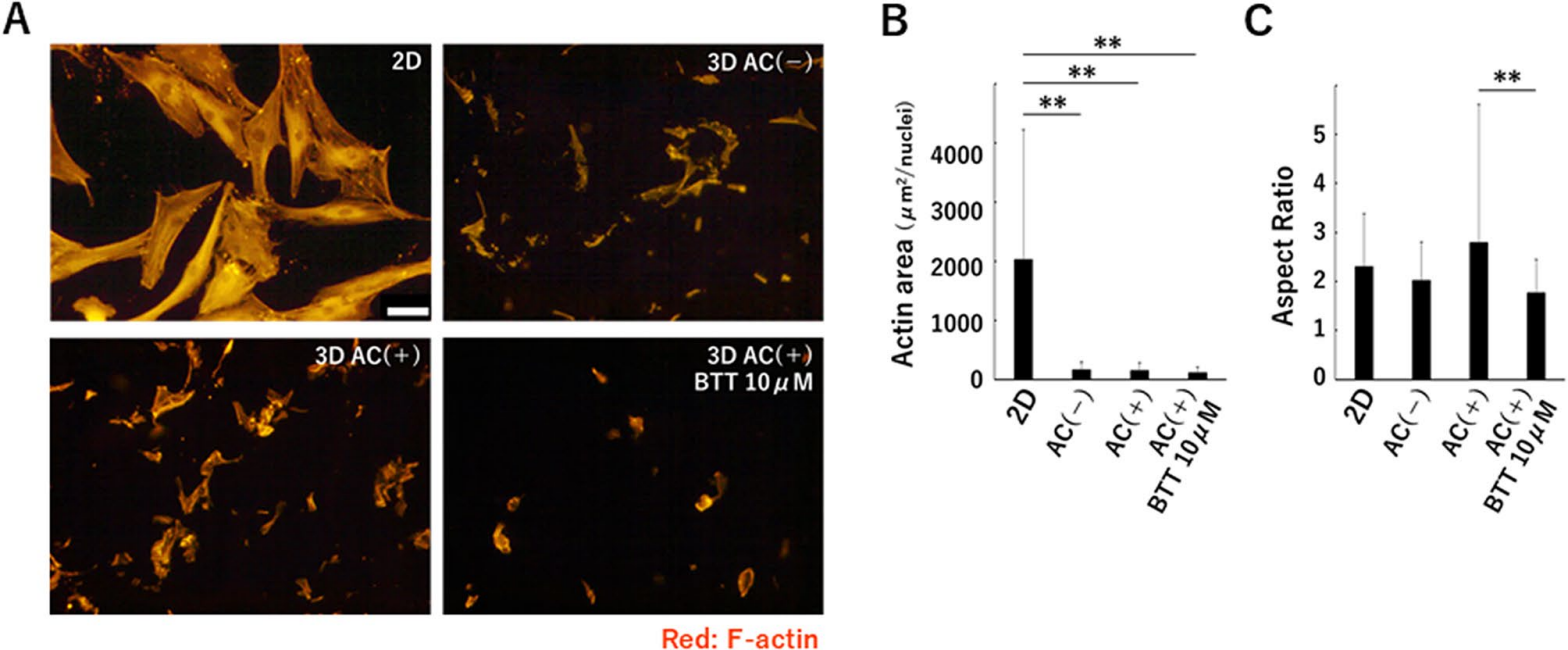
BTT 3033 alters the cell shape in the AC(+) group. (**A**) Visualization of actin filament in the cells of the 2D group, the AC(−) without BTT 3033 group, the AC(+) without BTT 3033 group, and the AC(+) with BTT 3033 group using fluorescent phalloidin. (**B**,**C**) To quantify the effect of BTT 3033 on cell shape, the cellular area (**B**) and aspect ratio (**C**) were calculated as described in the Materials and Methods. Scale bar: 50 μm. *p < 0.05, **p < 0.01.

The expression of *ITGA2* was significantly upregulated in the presence of BTT 3033 (10 μM) in all three groups (Figs. 5A, S3A, and S4A). Regarding other collagen-binding integrins, the expression of *ITGA10* was significantly promoted in the AC(+) group and the expression of *ITGA1* and *ITGA11* was significantly downregulated in the 2D culture group. In the AC(−) group, no other collagen-binding integrins showed significant change in the presence of BTT3033. Quantification of the cellular DNA on day 4 of culture showed that BTT 3033 (10 μM) negatively affected cell proliferation in the AC(+) group, but not in the 2D culture and the AC(−) group (Figs. 5B, S3B, and S4B). On day 4 of culture, cyclin D1 and p21 were highly expressed in the AC(+) group and the 2D group, but not in the AC(−) group in the presence of BTT 3033 (Figs. 5C, S3C, and S4C). Concerning the expression of MMP family, there were significant changes in the 2D culture and the AC(+) group, and there was no change in the AC(−) group. The change was particularly large in the AC(+) group, with MMP13 being significantly down-regulated in the presence of BTT3033, while MMP1 and MT-MMP1 were significantly up-regulated (Fig. 5D). As for the cell shape, cells in 3D culture were smaller than those in 2D culture (Fig. 6A,B) and cells in the AC(+) group cultured in the presence of BTT 3033 had a lower aspect ratio than cells cultured without BTT 3033 (Fig. 6A,C).

### α2β1 integrin affects the response to cyclic compressive loading stimulation in 3D culture

**Figure 7:**
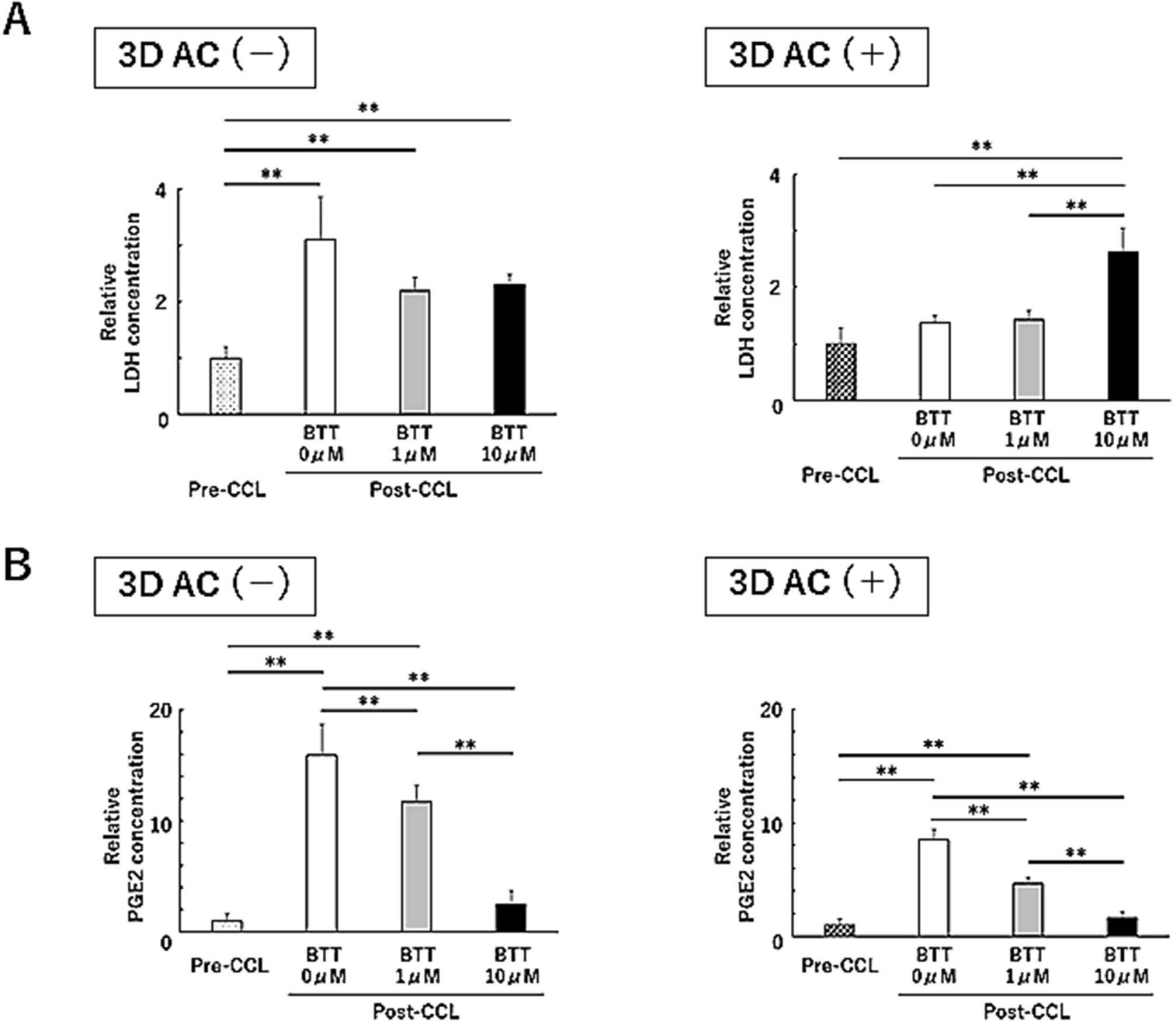
α2β1 integrin is involved in the mechanotransduction of cyclic compressive loading stimulation in three-dimensional culture cells. Concentration of LDH (**A**) and PGE2 (**B**) in the culture medium before and after cyclic compression loading stimulation (CCL) were compared. A representation of three independent experiments, each with three subjects is shown. *p < 0.05, **p < 0.01.

Cell–ECM interactions are thought to play a role in the response to mechanical stimulation^20^. 3D culture cells were subjected to cyclic compressive loading (CCL) stimulation using a bioreactor. First, we examined the concentration of lactate dehydrogenase (LDH) in the culture supernatant as a marker of cytotoxicity (Fig. 7A). In the AC(−) group, the concentration of LDH after CCL was significantly higher than that before CCL. This difference was also observed in the presence of BTT 3033. In the AC(+) group, however, the concentration of LDH did not increase in response to CCL, although a significant increase was observed in response to CCL in the presence of BTT 3033 (10 μM).

We also examined the effect of CCL on the production of prostaglandin E2 (PGE2), a well-known pathogenic molecule related to OA development (Fig. 7B)^21^. In a previous report, we showed that CCL on a 3D culture of human synovial fibroblasts significantly upregulated PGE2 production^22^. In both the AC(+) and AC(−) groups, CCL significantly increased the production of PGE2. This effect was suppressed in a dose-dependent manner by BTT 3033, and a complete suppression was observed at a concentration of 10 μM. When comparing the AC(+) and AC(−) groups, the degree of increase in PGE2 production was more prominent in the AC(−) group, although the effect of BTT 3033 was similar in both groups.

## Discussion

In the current study, several lines of evidence suggest that the interactions between atelocollagen gel and human articular cartilage-derived chondrocytes play an important role in various cellular functions. First, genome-wide differential gene expression analysis revealed that atelocollagen gel regulates the expression of cell membrane- and extracellular matrix-related genes, while the amount of atelocollagen gel in culture gradually decreased with time. Second, we found that the expression of *ITGA2*, which encodes alpha-2 integrin, was significantly increased in the presence of atelocollagen gel, and that inhibition of the α2β1 integrin pathway affected the cell shape and proliferation, the transcription of MMP family members, and the effect of mechanical stress on human articular cartilage-derived chondrocytes in 3D culture.

Previous studies have shown that cells grow healthily when cultured in collagen gel^23, 24^. Consistent with this, human articular cartilage-derived chondrocytes cultured in atelocollagen have been shown to proliferate and synthesize extracellular matrix in a cell density-dependent manner^25^. In the present study, we compared the growth of 3D cultured cells seeded onto atelocollagen sponges in the AC(+) and AC(−) groups, and found no significant difference in the total DNA content between the two groups over the course of a 4-week culture period. Previous studies using the same atelocollagen sponge have reported that embryonic stem cells showed good growth in 3D culture^26^, and similar findings have been reported for induced pluripotent stem cells^27^. While these previous studies used different cell types than the present study, the findings are consistent with ours in that atelocollagen gel does not interfere with cell proliferation. On the other hand, cell metabolic activity assessed using the Cell Counting Kit-8 appears to differ depending on the presence of atelocollagen gel^28^.

Atelocollagen is a naturally occurring collagen obtained by removing telopeptides from type 1 collagen and it is biodegradable^12^. Immunostaining and the measurement of collagen degradation products suggest that the atelocollagen gel and sponge are biodegradable. MMP family members are known to function as important elements of collagen degradation^12^. In the present study, the expression of MMP2, MMP3, MMP9, and MMP13 in human articular cartilage-derived chondrocytes increased in the AC(+) group. Among these MMPs, MMP13, which showed the greatest expression change, was classified as a collagenase and has a broad and strong proteolytic ability^29, 30^. MMP13 plays a role in nascent bone growth, remodeling, and in the maintenance of articular cartilage homeostasis, and is also recognized as a major MMP overexpressed in osteoarthritis^31, 32^. MMP9 was confirmed to have significant transcription promotion and an increased protein content in the AC(+) group. Similar to MMP13, MMP9 is also reported to have an increased expression in OA cartilage and to function as a cartilage-destroying factor. It is unclear which of these MMP families plays a central role, but it is likely that the microenvironment of cells differs significantly between early and late cultures using atelocollagen due to the enzymatic function of promoted MMPs. Chondrogenic markers were more highly expressed in cells of 3D cultures than in 2D culture, particularly in the early stages of culture, when atelocollagen gel is abundantly present. This result is consistent with a previous report showing that collagen gel contributes to both the differentiation and dedifferentiation of chondrocytes^33–35^. These findings collectively suggest that the presence of atelocollagen gel modulates gene expression in human articular cartilage-derived chondrocytes in 3D culture, leading to changes in the cell properties, such as in cell metabolic activity.

Consistent with previous reports, we found that dimensionality had a major impact on cells, with differences being observed between 2 and 3D cultures^36–38^. Genome-wide gene expression analysis also revealed that the presence of atelocollagen gel promotes the transcription of genes related to cell membrane–ECM interactions, particularly those related to growth factor, cytokine, and MMP activity. Among these molecules, *ITGA2*, a gene involved in cell–ECM interactions, was highly expressed in human articular cartilage-derived chondrocytes in the AC(+) group. 3D culture of human skin fibroblasts with type I collagen gel has been shown to induce integrin-mediated MMP13 expression, and p38 MAPK (mitogen-activated protein kinase) activation is important in this process^39^. Similarly, in the present study, atelocollagen gel induced the expression of MMP13, likely via integrin α2β1. These results, together with the results of the integrin α2β1 inhibition experiments, strongly suggest that *ITGA2* is a key molecule that mediates the effect of atelocollagen gel on human articular cartilage-derived chondrocytes. *ITGA10*, another collagen-binding integrin, showed a similar expression pattern as *ITGA2*. Although *ITGA10* is known to have an important function in endochondral ossification during development, it is suggested that it may also have a function related to cell events during cell culture using atelocollagen, as well as *ITGA2*. The functions and interactions of *ITGA2* and *ITGA10* should be verified in the future. Inhibition of integrin α2β1 resulted in the suppression of MMP13 expression, while significantly increasing the expression of MMP1 and MT-MMP1. From the perspective of the function of the entire MMP family, this can be regarded as a homeostatic reaction of the living body. Elucidation of that feedback mechanism may provide important insights in this area. In previous studies using *ITGA2* KO mice, new blood vessels were generated in a wound healing model^40, 41^ and degeneration was suppressed in an osteoarthritis model^42^, and alterations in the expression pattern of MMP family of genes have been confirmed. These phenotypes are consistent with the changes in the expression of MMP family members observed in the present study.

The cellular response of articular cartilage to mechanical stress, in the form of CCL, has been vigorously studied^43, 44^. In the present study, CCL was cytotoxic to human articular cartilage-derived chondrocytes in the AC(−) group and promoted PGE2 production. The involvement of the α2β1 integrin pathway in this cellular response was confirmed using the specific α2β1 integrin inhibitor, BTT 3033. In contrast, this cytotoxic reaction was not observed in the AC(+) group, and the production of PGE2 was also less pronounced in the AC(+) group than in the AC(−) group. Further studies on the potential role of integrin as a mechanoreceptor and the effect of mechanical stress on cultured cells are warranted.

Regenerative medicine and cell therapy, such as ACI, are gaining importance in the treatment of many diseases^45^. Although further investigation is necessary, our present findings have implications in ​regenerative medicine for articular cartilage disorders. Since collagen is an ECM protein of major importance in many cell types and MMP family members function as key regulators of ECM under both physiological and pathological conditions, our findings likely have implications not only in articular cartilage disorders, but also in other disorders in which cell–ECM interactions play an important role.

## Materials and methods

### Ethics statement

This study was conducted using cells derived from human articular cartilage and was approved by the Osaka University Institutional Ethical Committee (approval ID 16085-4). Written informed consent was obtained from all subjects, and all methods were performed in accordance with the relevant guidelines and regulations.

### Scaffolds

Commercially available atelocollagen sponge (Mighty, KKN-CSM-50, Koken, Tokyo, Japan) and 1% atelocollagen gel (Koken) were used. Atelocollagen primarily consists of type I collagen derived from bovine dermis. The sponge has an interconnected pore size of 30–200 μm and can withstand a compressive loading of up to 40 kPa.

### Isolation of human articular cartilage-derived chondrocytes and 2D culture

Human knee articular cartilage was aseptically obtained from eight patients aged 58–83 years who underwent total knee arthroplasty to treat osteoarthritis. The cell isolation protocol was essentially the same as previously described^22^. Briefly, cartilage specimens were rinsed with phosphate-buffered saline (PBS), minced meticulously, and digested with 0.1% collagenase in Dulbecco Modified Eagle’s Medium/high glucose (DMEM, D6429) (Sigma-Aldrich, St. Louis, MO, USA) with 1% penicillin/streptomycin (Thermo Fisher Scientific Cat# 15140122) for 6 h at 37 °C. Cells were cultured in DMEM with 10% fetal bovine serum (FBS, Lot# 174012) (Nichirei Bioscience, Tokyo, Japan) and 1% penicillin/streptomycin using commercial tissue culture polystyrene (TCPS) dishes or plates and used at passages two to four.

### 3D culture in atelocollagen sponges

Cells were seeded onto atelocollagen sponges using two methods. For 3D culture with atelocollagen, cells (1–5 × 10^5^/sponge) were suspended in growth medium and mixed with an equal volume of 1% atelocollagen gel to produce a cell suspension in a 0.5% collagen solution. The solution was carefully dripped onto the sponge and allowed to soak. For the 3D culture without atelocollagen, cells suspended in growth medium were seeded onto the sponge using the same procedures, but without prior mixing with 1% atelocollagen gel.

### Histological analysis

Cells in sponges were fixed with fresh 4% paraformaldehyde (paraformaldehyde phosphate buffer solution, 163-20145) (Wako Pure Chemicals, Osaka, Japan) at 4 °C for 2 h and then incubated overnight with a 30% sucrose/PBS solution. After embedding with optimal cutting temperature compound, cryosections (10 μm) were prepared for hematoxylin and eosin (H & E) staining and immunostaining. After blocking with Blocking One Histo (06349-64, Nacalai Tesque) in 1× PBST (1× PBS, 0.1% Tween 20) at room temperature for 1 h, the sections were incubated at 4 °C overnight with the following primary antibodies: goat anti-type I collagen (1:200; 1310-01, Southern Biotech), rabbit anti-vimentin (1:400; ab92547, Abcam), rabbit anti-MMP1 (1:200; ab52631, Abcam), mouse anti-MMP2 (1:200; ab86607, Abcam), rabbit anti-MMP9 (1:200; ab38898, Abcam), and rabbit anti-MMP13 (1:200; ab39012, Abcam). For anti-type I collagen and anti-MMP9 antibodies, immune complexes were detected using anti-goat IgG H & L (HRP) (1:500; ab97110, Abcam) and ImmPACT DAB (SK-4105, Vector Laboratories). For anti-vimentin, anti-MMP1, anti-MMP2, anti-MMP9, and anti-MMP13 antibody, immune complexes were detected using anti-Rabbit IgG (H + L) Alexa Fluor 488 (1:400; A-21206, Invitrogen), or anti-mouse IgG2a Alexa Fluor 488 (1:400; A-21131, Invitrogen). The actin cytoskeleton was detected using Acti-stain 555 Fluorescent Phalloidin (PHDH1, Cytoskeleton Inc., Denver, CO). Images were obtained using a DMi8 (LEICA) microscope or a Keyence BX-X700 microscope.

### Metabolic activity assay

Cell metabolic activity was assessed using a Cell Counting Kit (CK04-01, Dojindo Molecular Technologies) according to the manufacturer’s instructions with some modifications. For the comparison between the AC(−) and AC(+) groups, cells were seeded onto an atelocollagen sponge at a density of 1.5 × 10^5^ cells/sponge and then incubated in a 96-well plate for 1, 8, 22, and 29 days. For the comparison between the 2D and 3D culture groups, cells were seeded onto a 96-well plate directly at a density of 5 × 10^3^ cells/well or onto an atelocollagen sponge at a density of 5 × 10^3^ cells/sponge, and then incubated for 1, 2, 4, 5, 6, and 7 days. At each time point, 10 μL of solution from the Cell Counting Kit-8 (CCK-8; Dojindo, Kumamoto, Japan) was added to each well and incubation was continued in a 5% CO_2_ atmosphere at 37 °C for 1 h. Absorbance at 450 nm was measured using a microplate reader (MultiSkan GO; Thermo Fisher Scientific, Inc.).

### DNA content measurement

Cellular DNA was extracted using a PureLink Genomic DNA Purification kit (Thermo Fisher Scientific, United States) according to the manufacturer’s instructions. For the cell proliferation assay, cells were seeded onto an atelocollagen sponge at a density of 1.5 × 10^5^ cells/sponge and then incubated on a 96-well plate for 1, 8, 14, and 29 days. At each time point, samples were harvested for treatment with a digestion buffer and lysates were used for DNA extraction. DNA concentration was determined fluorometrically using a Qubit 4.0 Fluorometer (Thermo Fisher Scientific).

### RNA extraction and quantitative real-time RT-PCR

Total RNA was extracted using Trizol (Invitrogen) and a PureLink RNA Purification kit (Thermo Fisher Scientific), and reverse transcribed into cDNA using a High-Capacity RNA-to-cDNA kit (Thermo Fisher Scientific). Quantitative PCR was performed using Power SYBR Green Master Mix and QuantiStudio 7 Pro Real-Time PCR System (Thermo Fisher Scientific), according to the manufacturer’s instructions. Nucleotide sequences of primers are described in Table S1. The expression of the target genes was normalized to that of the reference gene, glyceraldehyde-3-phosphate dehydrogenase (*GAPDH*).

### Measurement of hydroxyproline in cell culture supernatants

We performed a colorimetric method using the QuickZyme kit (QuickZyme Biosciences, Leiden, the Netherlands) for hydroxyproline measurement. In brief, the supernatant on day 3 of culture was harvested and hydrolyzed in 6 M HCl in a safety centrifuge tube for 16 h at 95 °C. Reconstituted samples were centrifuged for 10 min at 13,000×*g*, and the supernatants were used for the measurement according to the manufacturer's protocol.

### Microarray analysis

Experiments were conducted in triplicates for all three culture conditions (2D culture, 3D culture with atelocollagen gel, and 3D culture without atelocollagen gel). Cells in the growth phase in 2D culture or cells on day 4 of 3D culture were harvested. The isolated total RNA was amplified and labeled with Cy3 using a Low Input Quick Amp Labeling Kit (Agilent Technologies). Hybridized arrays were scanned using an Agilent DNA Microarray Scanner (G2565CA). Microarray analysis was carried out using Takara Bio. Data analyses were carried out using the Subio platform version 1.24 (Subio, Inc., Kagoshima, Japan, http://www.subio.jp). Microarray datasets are available at the National Center for Biotechnology Information Gene Expression Omnibus (GEO) database (Accession no. GSE 154723).

### Cell shape analysis

Samples were stained with 4′ 6‐diamidino‐2‐phenylindole (DAPI) and phalloidin to show the nuclei and F-actin filaments, respectively. Fluorescence microscopy images were obtained using a BZ-X 700 fluorescent microscope system (Keyence, Osaka, Japan). For cell shape analysis, randomly selected cells (more than 60 cells per group) were used. Aspect ratio analysis was performed using a Hybrid Cell Counting application (Keyence) by dividing the major axis by the minor axis of cells.

### Cyclic compression loading stimulation

Cyclic compressive loading was applied to sponges using a custom-designed apparatus, a cyclic load bioreactor (CLS-5J-Z, Technoview, Osaka, Japan), as previously described^22, 46, 47^. Briefly, cells were cultured in sponges on 96-well culture plates and a cyclic load compressive load of 40 kPa was applied to the sponges for 1 h at a rate of 0.5 Hz using metal pistons. Medium replacement was performed 12 h before, immediately before and after, and 12 h after stimulation. The collected culture supernatant was used to measure the protein concentration.

### In vitro cytotoxicity assay

The amount of LDH released from the cells was measured using a Cytotoxicity LDH Assay Kit-WST (CK1205, Dojindo Molecular Technologies) according to the manufacturer's protocol. The culture supernatant 12 h before stimulation and the culture supernatant 12 h after stimulation were compared, and the ratio of the amount of protein contained was calculated.

### Quantitative PGE2 protein analysis

An enzyme immunoassay was performed to measure the concentration of PGE2 in the culture supernatant using a HTRF human PGE2 assay kit (CIS Bio International, Saclay, France). The culture supernatant 12 h before stimulation and the culture supernatant 12 h after stimulation were compared, and the ratio of the amount of protein contained was calculated.

### Statistical analysis

Data are presented as means and standard deviations (SDs). Single comparisons were performed using Student’s t test, and multiple comparisons were performed using one-way analysis of variance (ANOVA) and a post hoc Tukey–Kramer test. Statistical analyses were performed using Ekuseru–Toukei (Social Survey Research Information Co., Ltd., Tokyo, Japan) and a *p* < 0.05 was considered statistically significant.

## Supplementary Information

Supplementary Information.
