# Supplementary material for: Integrin α2β1 plays an important role in the interaction between human articular cartilage-derived chondrocytes and atelocollagen gel
Source: Sci Rep. 2021 Jan 19;11:1757. doi: 10.1038/s41598-021-81378-2 (PMC7815876; doi:10.1038/s41598-021-81378-2)
Supplement: Supplementary file 1 — Supplementary Information. [file 41598_2021_81378_MOESM1_ESM.pdf]

The title of the manuscript:

Integrin α2β1 plays an important role in the interaction between human articular cartilage-derived chondrocytes and atelocollagen gel

Authors:

Takashi Kanamoto[1], Minami Hikida[1], Seira Sato[1], Shohei Oyama[2], Yoshihito Tachi[3], Sanae Kuroda[4], Takeo Mazuka[5], Kosuke Ebina[2], Tsuyoshi Nakai[4], Ken Nakata[1]

The corresponding author:

Name: Takashi Kanamoto

E-mail: takanamoto@hss.osaka-u.ac.jp

The affiliation and address of the authors:

[1]Department of Medicine for Sports and Performing Arts, Osaka University Graduate School of Medicine, 2-2 Yamadaoka, Suita, Osaka 565-0871, Japan

[2]Department of Musculoskeletal Regenerative Medicine, Osaka University Graduate School of Medicine, 2-2 Yamadaoka, Suita, Osaka 565-0871, Japan

[3]Olympus-RMS CORP., 3-1-7 Myojin-cho, Hachioji, Tokyo, 1920046, Japan

[4]Department of Orthopedic Surgery, Itami City Hospital, 1-100 Koyaike, Itami, Hyogo, 664-8540, Japan

[5]Department of Orthopedic Surgery, Hannan Chuo Hospital, 3-3-28 Minami-shinmachi Matsubara, Osaka 580-0023, Japan

Primer	Sequence	Primer	Sequence
CDK4 F	ATGGCTACCTCTCGATATGAGC	*MMP1 F*	CCCAAAAGCGTGTGACAGTAAG
CDK4 R	CATTGGGGACTCTCACACTCT	*MMP1 R*	CTTCCGGGTAGAAGGGATTTG
CDK6 F	TCTTCATTCACACCGAGTAGTGC	*MMP2 F*	GCACCCATTTACACCTACACCAA
CDK6 R	TGAGGTTAGAGCCATCTGGAAA	*MMP2 R*	AGAGCTCCTGAATGCCCTTGA
Cyclin D1 F	GCTGCG AAGTGGAAACCATC	*MMP3 F*	CGTGAGGAAAATCGATGCAG
Cyclin D1 R	CCTCCTTCTGCACACATTTGAA	*MMP3 R*	CTTCAGCTATTTGCTTGGGAAAG
p21 F	GCCCAGTGGACAGCGAGCAG	*MMP9 F*	AGTCCACCCTTGTGCTCTTC
p21 R	GCCGGCGTTTGGAGTGGTAGA	*MMP9 R*	TTTCGACTCTCCACGCATC
COL1 F	GTCGAGGGCCAAGACGAAG	*MMP13 F*	CTTCCCAACCGTATTGATGC
COL1 R	CAGATCACGTCATCGCACAAC	*MMP13 R*	ACTTCTTTTGGAAGACCCAGTTC
COL11 F	GCTTTTCCCAGGAGGATTTC	*MT1-MMP1 F*	TCAGGGCAGTGGATAGCGA
COL11 R	GAGAGTCAGGAGGGGAGCTT	*MT1-MMP1 R*	GCCGGTTCTACCTTCAGCTTC
SOX9 F	TACGACTACACCGACCACCA	*ITGA1 F*	CAGCCCCACATTTCAAGTCGT
SOX9 R	TCAAGGTCGAGTGAGCTGTG	*ITGA1 R*	ACCTGTGTCTGTTTAGGACCA
Aggrecan F	ACAGCTGGGGACATTAGTGG	*ITGA2 F*	GCAACTGGTTACTGGTTGGTT
Aggrecan R	GTGGAATGCAGAGGTGGTTT	*ITGA2 R*	GAGGCTCATGTTGGTTTTCATCT
Proteoglycan4 F	TCCATTCAGTCCACCATCTCC	*ITGA5 F*	GCCTGTGGAGTACAAGTCCTT
Proteoglycan4 R	TGTCCAGTTAGTCCTCCAAATCCT	*ITGA5 R*	AATTCGGGTGAAGTTATCTGTGG
Ihh F	CATTGAGACTTGACTGGGCAAC	*ITGA10 F*	CTTCAGTTCTGGGATATGTGCC
Ihh R	AGAGCAGGCTGAGTTGGGAGTCGC	*ITGA10 R*	CCAGTCTTCGTAGGAAGGTCT
Runx2 F	CAGACCAGCAGCACTCCATA	*ITGA11 F*	TCACGGACACCTTCAACATGG
Runx2 R	CAGCGTCAACATCATTC	*ITGA11 R*	CCAGCCACTTATTGCCACTGA
HIF-1α F	CTGACCCTGCACTCAATCAA	*GAPDH F*	TCTCTGCTCCTCCTGTTCGAC
HIF-1α R	TCCATCGGAAGGACTAGGTG	*GAPDH R*	GTTGACTCCGACCTTCACCTTC
HIF-2α F	TGTGTGAACCAATCCAGCAC		
HIF-2α R	ACTTCATGTCCATGCTGTGG		

Supplementary Table S1. Primer Sequences, Related to Figures 1, 2, 4, 5, and S2-4

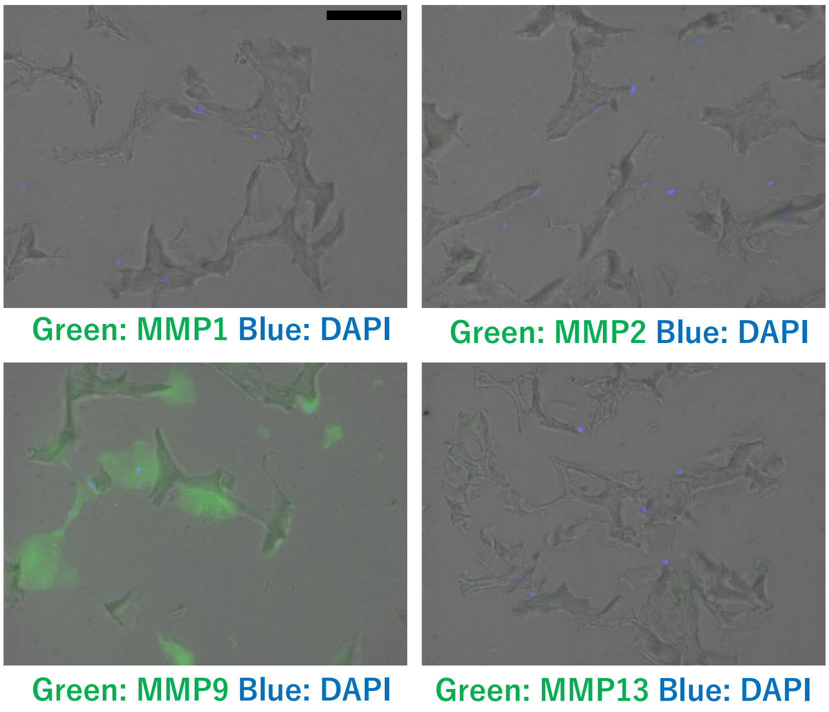

Supplementary Figure S1. Expression of MMP proteins in AC (+) culture group

Representative images of MMPs (green) immunostaining in AC (+) culture sample on day 4 culture. Blue: DAPI. Scale bar: 50 µm.

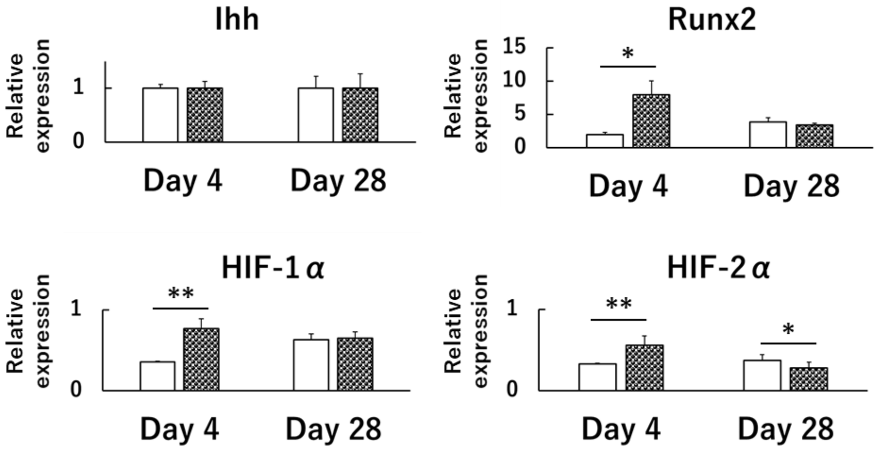

Supplementary Figure S2. Expression of hypertrophic markers and hypoxia inducible factors

One representative of two independent experiments, each with three subjects, is shown. Normalized expression relative to 2D culture is shown. *p < 0.05, **p < 0.01.

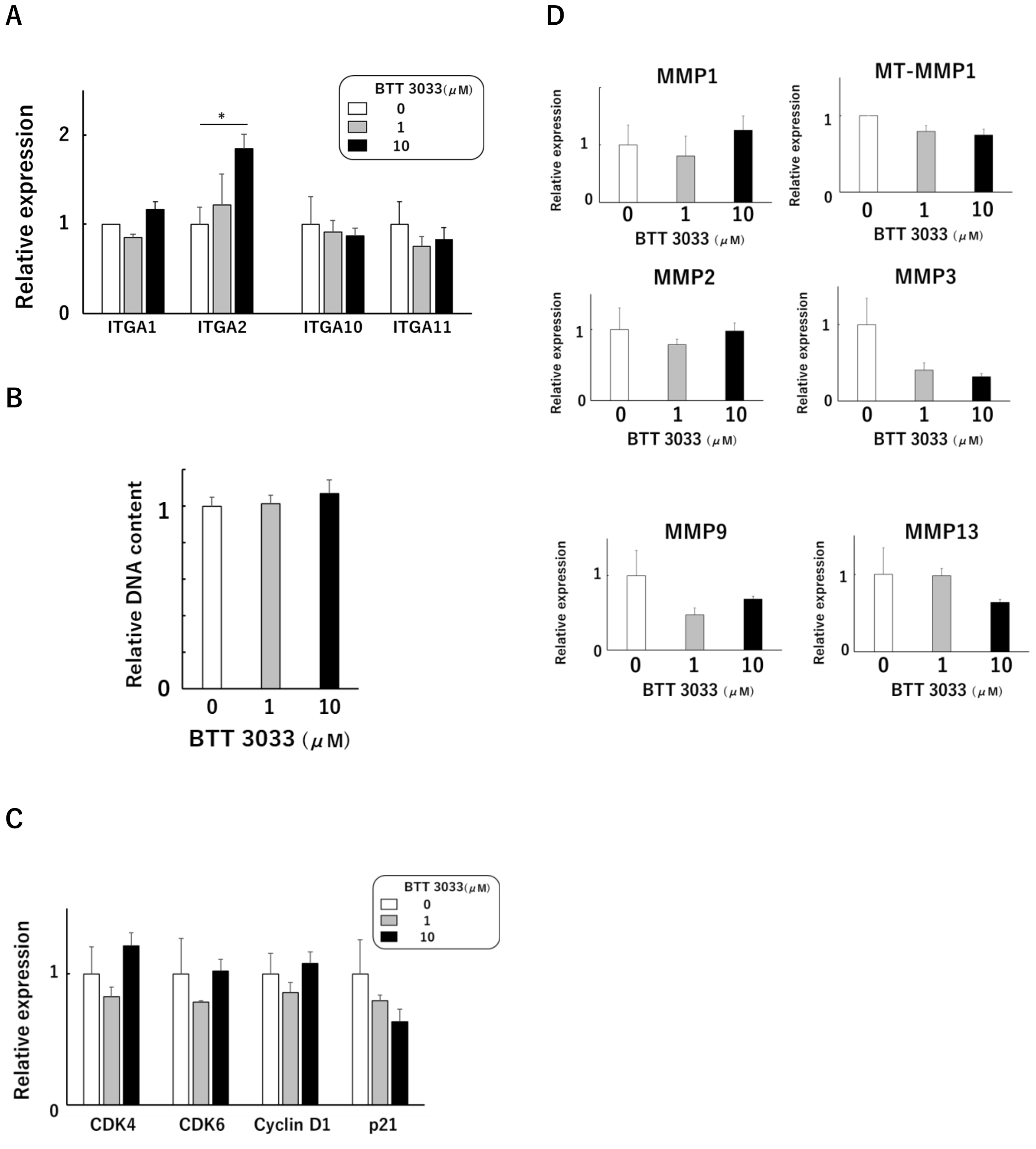

Supplementary Figure S3. The effect of BTT 3033 on AC (-) culture group. Expression of collagen-binding integrins (A), cell cycle-related factors (C), and MMP family (D) in the presence of BTT 3033, a selective inhibitor of α2β1 integrin, in cells cultured without atelocollagen gel. Normalized expression relative to cells cultured in the absence of BTT 3033 is shown (A, C, D). (B) DNA content relative to cells cultured in the absence of BTT 3033 is shown. $*p < 0.05$, $**p < 0.01$.

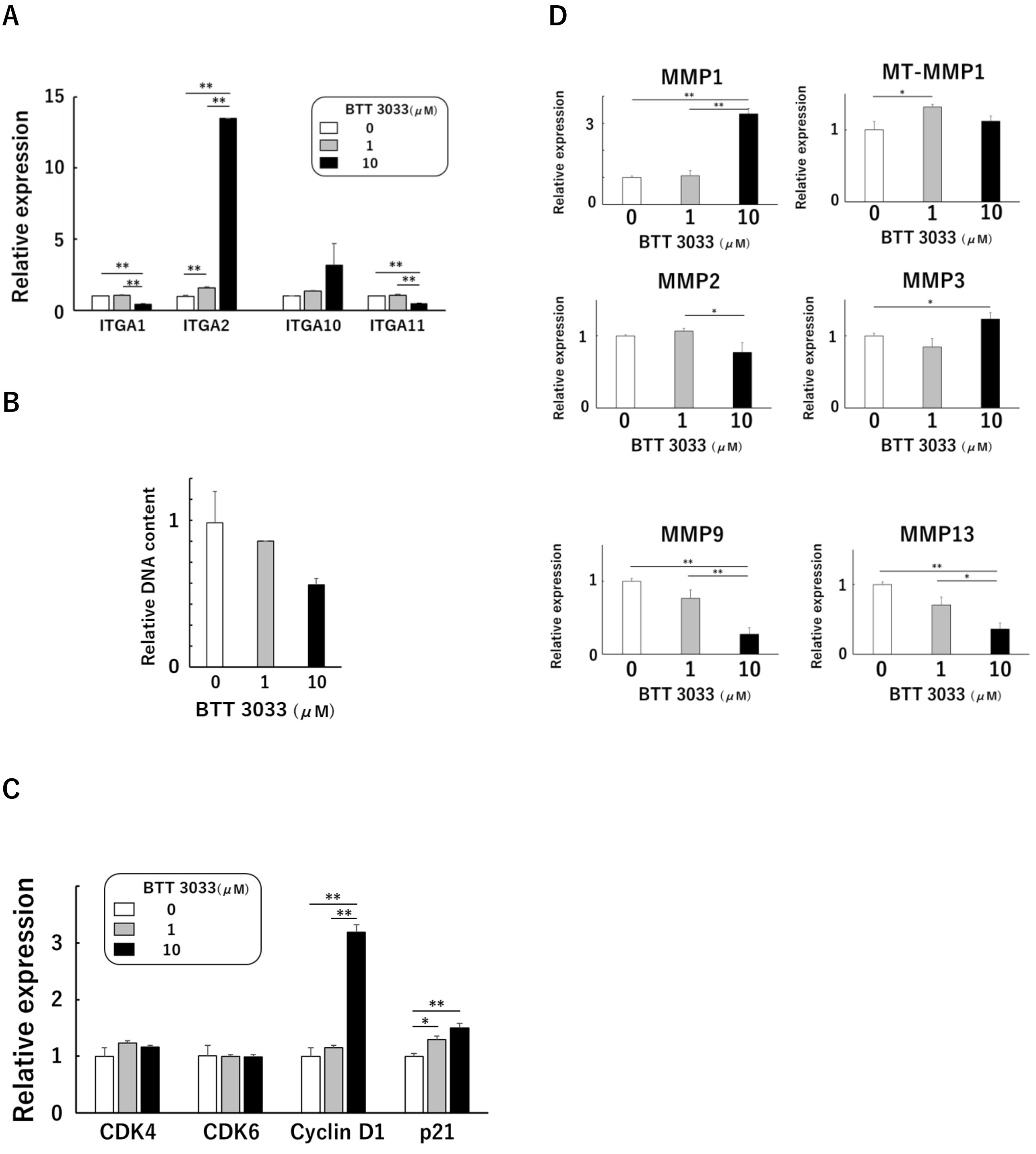

Supplementary Figure S4. The effect of BTT 3033 on 2D culture group. Expression of collagen-binding integrins (A), cell cycle-related factors (C), and MMP family (D) in the presence of BTT 3033, a selective inhibitor of α2β1 integrin, in cells cultured without atelocollagen gel. Normalized expression relative to cells cultured in the absence of BTT 3033 is shown (A, C, D). (B) DNA content relative to cells cultured in the absence of BTT 3033 is shown. *$p < 0.05$, **$p < 0.01$.
